# Lithotrity. The Baron Heurteloup's Instruments

**Published:** 1830-06

**Authors:** 


### LITHOTRITY.

*Lithotrity. The* Baron Heurteloup's *Instruments.*

At a meeting of the members of the Royal Institution, held on the 14th of May, the subject of lithotrity was introduced by Mr. Gilbert Burnett, and the various instruments used in that operation, with the methods of employing them, demonstrated and described; more especially referring to the improved apparatus of the Baron

Heurteloup. As the mechanism of these latter had not previously been made public, although their action had frequently been shown, an abstract of the detail given by Mr. Burnett (which was illustrated by enlarged diagrams of the parts of the several instruments, both separate and in conjunction,) will doubtless be interesting to the profession.

Mr. Burnett avoided (and we think wisely) entering at all into the controversy which has been, and still is, carrying on with respect to the originality of the process, and the inventors of its several parts; and confined his observations chiefly to what is of more essential importance, the operation itself, and the instruments with which it can be most effectually and easily performed: for, as he observed, it is a far more grateful task to show how much surgery stands indebted to Gruithuisen, Civiale, Le Roy, and Heurteloup, as colleagued in the advancement of lithotrity and the improvement of its apparatus, than as rivals contending for a prize which would be incomplete if deprived of the discoveries of either.

It is a matter of notoriety, that in females the urethra may be so far dilated that stones of considerable size can be extracted from the bladder; and in the male, small calculi are sometimes voided in the same manner, or even occasionally have been extracted by means of lengthened forceps. Now, lithotrity is an operation by which calculi of almost any size, at least to that of twenty-four lines in diameter, may be reduced to powder, or to fragments so small, that they can be washed out of the bladder by injections, or carried away in the ordinary flow of urine.

The elements of this operation hence are,

1. The reduction of the curves of the urethra to a right line, so that straight instruments may be passed there-through, and its capacity dilated.

2. The introduction of an apparatus, the different parts of which are fitted to sound, to seize, and also to destroy, the stone.

3. The injection of fluid into the bladder, by which, during the operation, its parietes are kept from coming in contact with the instruments, and by which the detritus may be afterwards washed out.

These elements of lithotrity may claim in part a very early date; but, although long known, it was not till lately that they became of much practical importance; and to the distinguished foreigners already mentioned, is the credit due of combining them in one efficient system, as well as of constructing a series of very ingenious instruments, by which the operation not only can be, but has been frequently performed with success.

The simplest form of apparatus, and that which is found most useful for the destruction of small calculi, consists of a straight canula, containing within it a three-branched forceps, the elasticity of which causes its tentacles to expand when pushed beyond the end of the canula by which they are concealed; or, rather, when the canula is withdrawn, so as to permit their developement. The ends of the forceps are curved, and all of different lengths, so that they cannot include any fold of the bladder, even were it to contract or fall upon them; they are all united at their manual extreme, forming by their conjunction a tube through which a drill (the length of which is regulated by the length of the forceps, so that it cannot injure the bladder, and the crown of which may be either eccentric or not, according to the size of the calculus,) acts, and grinds away the stone: if the stone be small, it may at once be crushed by the pressure of the drill, when held firmly by the forceps; or, when bored through once, it need not be let entirely to slip from the forceps in order to make another perforation; for, if the manual end of the instrument be tapped while the branches are slightly relaxed, a fresh surface will be presented, which may be ascertained by the resistance afforded to the drill. At the side of the canula is a foramen, with a screw or plug, by loosening which the fluid may be allowed to escape.

For the destruction of stones exceeding eight lines in diameter, this instrument is tedious; and, when repeated sittings are required, it might so happen that one branch of the forceps might enter a perforation in the stone, and occasion much difficulty in the operation, if not danger to the patient. For the destruction of stones from eight to fourteen lines in diameter, the crown of the drill has been enlarged, so that they may be reduced at once to fragments; and this has been done in various modes, the most efficient of which would seem to be the projection of a small branch, somewhat resembling a comma, from the side of the crown of the drill, after a perforation has been begun, so that it is rendered still more eccentric in its action than the mere eccentric drill: this projecting branch, or comma, can be protruded or withdrawn at pleasure, by a rod which runs through the centre of the drill. The forceps and drill, thus modified, have been named "Instrument à trois branches et à virgule." The invention of the previous instruments we believe to rest with Gruithuisen, Civiale, and Le Roy; but they have each been improved by Baron Heurteloup in design and construction. The following, which are well fitted to seize and destroy stones exceeding fourteen lines in diameter, and those which are flat, we are informed belong solely to the latter.

It is a fact well known that, when the stone is large, the bladder in general is small; not only relatively, but absolutely smaller than when the stone is of moderate dimensions; and furthermore, that the bladder contracts frequently upon the stone, and assumes a preternatural muscularity, the inner surface having not merely a rugose, but a columnar character. This, of course, will prevent that viscus from being freely distended by injection, will keep the stone nearer the neck of the bladder, and might interfere with the fully extended tentacula of the three-branched forceps. The larger the calculus is, the more numerous, of course, must be the perforations made in it before its substance will break down, and, even with the eccentric or virgule drills, the process would still be tedious, although much more speedy than if simple perforations alone were made. The irregularity of stones likewise gives rise to difficulty in their apprehension and firm retention, as it seldom happens that all the claws, moving simultaneously, close upon the calculus, especially if it be a large one; so that, when pressed upon by the drill, it is apt to slip or to elude the grasp. Furthermore, that form of the three-branched forceps previously described, the whole of the tentacula of which are in union, and of necessity move together, can never, at its utmost stretch, project a span of more than one third of its circumference; and even this diminishes rapidly towards the canula, there being no means of keeping the springs at any greater width from each other.

To overcome these difficulties, all the instruments have been modified and others added by Baron Heurteloup, who designates this improved apparatus, which is fitted to destroy stones twenty-four lines in diameter, "Evideur à forceps," *(excavator with forceps,)* and it consists of several parts, which may briefly be thus described: The straight canula, instead of being a simple cylinder, as in the other apparatus, contains within it another tube, nearly equal to itself in length, which is four-sided, so that the chief channel is subdivided into five compartments, a square central one and four circumferential passages, through each of which latter passes one branch of the forceps, which here consists of four instead of three tentacula. Three of the branches are terminated at their vesical extremes by simple curves, the fourth and longest by a grooved knob, which closes in the whole, and facilitates the introduction. The four branches of the forceps being separate from each other, and sliding in separate grooves, may hence, by knobs fixed to their manual ends, be each moved independently of the other, or, by a case which encloses the knobs of all, be made to act simultaneously; thus combining, with all the advantages of the common forceps, the very important privilege of separate and more or less conjoint action. The four-branched instrument, by the withdrawal of one of its tentacles, may also be converted into a superior three-branched forceps, as the span of its branches will be the diagonal of its square, instead of the mere base of an equilateral triangle, as in the former case; so that stones of much greater magnitude can be seized, and with much less extension of the instrument: furthermore, the knobbed tentacula, when withdrawn, will still more widen the grasp of the other three, and keep them fully and firmly apart. The separate motions of the tentacles will also allow each to be separately pulled on, so that every one can be firmly acting on the stone; a very important circumstance. The knobbed tentacle may, subsequently to the prehension of the stone, be detruded, and it will also close upon it; and, when all four branches act, the retention is the most firm that can be conceived. Through the central cavity, the subsidiary instruments are introduced: these consist of a simple steel rod, called the "Indicator," and a very delicate three-branched "assistant forceps," (*pince servante*,) designed to bring the calculus within the grasp of the larger and stronger instrument, should it not in the first instance be easily apprehended. The instruments destined to destroy the stone consist of a pointed steel red, "the perforator," which, rotated by a common drill bow, makes a hole in the stone; and the excavator (*evideur*), which is a somewhat similar rod, the end of which is a very powerful rasp or file, which can be inclined, by means of a screw, to any angle the size of the calculus may require, and which, when rotated, by means of the bow as before, excavates the stone, leaving nothing but a thin shell formed of its outer layers, which the withdrawal of the branches of the forceps will usually be sufficient to crush: should it, however, resist their force, the indicator may be used as a "percusseur," and one or two strokes of the simple steel rod upon the shell will inevitably reduce it to fragments.

Such is the economy of the excavating apparatus, which, for the destruction of large round stones, is incomparably the best instrument yet invented; but the seizure and destruction of flat stones, or the flattened shell-like fragments of larger calculi, have presented many difficulties to the operating lithotrist. The "shell-breaker," or *brise-coque*, would seem to obviate these. It has been said to be merely an improvement on the *brise-pierre* of Amusat: in truth, the chief difference between them is that, although both are ingenious instruments, the one is highly useful, the other inapplicable in practice.

The shell-breaker both seizes and crushes the calculi or fragments, without the assistance of drill or perforator. Hence it is necessarily made very powerful in its branches, and, to allow such strength in its construction, without increasing too much the size of the tube, it consists but of two branches, which entirely fill up the canula, and are terminated by strong roughened jaws, which open by a spring placed between them to receive the calculus when detruded from the canula, and, closing in their retreat, crush the hardest stone or fragment between their hawk-bill ends. Motion is communicated to these branches by a double rack and two wheels contained within the handle, the inward and outward progress being commanded by a spring catch which regulates the rack.

It has been usual to operate on an ordinary bed or couch, and to fix the lithotritic instruments in a spring vice held by an assistant, while the surgeon completed the perforation of the calculus by the drill and bow; but when a much more powerful mean, viz. the excavator, is employed, it becomes necessary that the instruments should be held more steadily, and for this purpose Baron Heurteloup has constructed a very ingenious bed, the "lit rectangle," to the front of which there is a movable vice attached, and which can also be lowered as a whole by movable hind legs, so as to form an inclined plane, and elevate the pelvis, &c. without disturbing the patient. This, however, as well as the catheter with foramina several inches down the side, and the syringe with bows, although useful instruments, cannot be deemed essential to the operation; and they will, of course, be adopted or rejected according to circumstances, of which the attending surgeon can alone be the proper judge.

When vaccination was first introduced, the officious zeal of a few intemperate supporters of the cause endangered the success of it by foolishly asserting that it was infallible; that smallpox never had, and never could, occur when the constitution had been protected by its influence. The public naturally began to doubt the merits of vaccination, when they found that they were deceived by studied misrepresentation; and they who were determined to oppose the introduction of the new practice, were assisted in their endeavours to prejudice the public mind against it, by incessant appeals to the want of candour of some of its partisans. In the same manner, the great surgical improvement of the present day, lithotrity, has been over-praised by those who, from being imperfectly acquainted with the subject, assert that the operation of lithotomy will henceforth be entirely forgotten.

This is by no means a fair and impartial view of the case, and those who are most deeply interested in the success of lithotrity, as Heurteloup, Civiale, &c. have shown their judgment by abstaining from such exaggerated encomiums. It must be granted that there are cases of calculus in the bladder which do not admit of the employment of the lithotritic apparatus; but the important and gratifying fact that the great majority of patients labouring under this formidable disease may now be rescued by the comparatively mild and perfectly safe operation of lithotrity, is established beyond dispute. Among those who have distinguished themselves in the lithotritic art, both by ingenious improvements in different instruments and great practical dexterity, the Baron Heurteloup claims a high rank. As a proof of his merit, the Academy of Sciences of Paris bestowed upon him their first surgical prize for his improvements of lithotritic instruments.

On Friday, the 22d, the lithotritic instruments invented by Mr. LUKIN, the American, and exhibited in this country and on the continent some years ago, were placed on the library table of the Royal Institution. They are certainly very inferior to those recently constructed; but if, as is stated, his were the first made, much credit is due to him for having so far advanced the design. They consist of a *straight* silver canula, concealing a four-branched forceps, terminated by watchspring loops, for the prehension and retention of the stone; and a small trephine drill, with several forms of chisels for its destruction; also a two-branched forceps, of very ingenious mechanism, but which seemed rather fitted to extract stones from the urethra, or very small fragments from the bladder, than to hold larger calculi whilst being reduced to pieces, or to act with the energy of a *brise-coque* or *brise-pierre*.

